# Venous malformation with localized intravascular coagulopathy in children treated with sclerotherapy and LWMH

**DOI:** 10.3389/fped.2026.1835885

**Published:** 2026-07-01

**Authors:** Xiao Gao, Xuming Wang, Yuhua Wei, Shuai Niu, Le Yang, Lei Xu

**Affiliations:** Department of Vascular Surgery, Shandong Provincial Hospital Affiliated to Shandong First Medical University, Jinan, China

**Keywords:** coagulation, localized intravascular coagulopathy, LWMH, sclerotherapy, venous malformations (VM)

## Abstract

**Introduction:**

Localized intravascular coagulopathy (LIC) is a common, underrecognized coagulation complication of pediatric venous malformations (VMs), driven by chronic thrombogenic activation within VM lesions. Developmental hemostatic traits in children may exacerbate LIC severity, but clinical characteristics and optimal management of pediatric LIC remain poorly defined.

**Methods:**

This retrospective cohort study included 116 pediatric VM patients with complete laboratory test results who underwent sclerotherapy at Shandong Provincial Hospital between August 2018 and August 2025. LIC was defined as D-dimer ≥0.60 μg/ml. Chi-square test, Fisher's exact test, Mann-Whitney U test, and correlation analysis were used to explore factors associated with LIC occurrence and severity.

**Results:**

Of 116 patients, 51 (44.8%) had LIC (38 mild, 11 moderate, 2 severe). No significant association was found between LIC (occurrence or severity) and sex, age, or anatomic location. All severe LIC cases occurred in extensive VMs involving ≥2 joint planes. Sclerotherapy combined with low-molecular-weight heparin (LMWH) rapidly corrected coagulation abnormalities in severe LIC.

**Discussion:**

LIC occurrence in pediatric VMs is independent of demographic or anatomic factors, while severity correlates directly with lesion burden. Sclerotherapy is the core intervention for LIC control, with LMWH serving only as perioperative adjuvant therapy. This study supports routine screening for occult VM in unexplained pediatric coagulopathy, providing real-world evidence for optimized clinical management.

## 1 Introduction

Venous malformation (VM), is one of the most common slow-flow congenital vascular malformation. While lower limbers or multiple lesions are affected in VMs, coagulation abnormality could be prominent especially simulated by fractures, immobilization, and sepsis, referred to as localized intravascular coagulopathy (LIC) (1–4). Severe LIC can induce DIC with release of pro-coagulant factors and consumption of coagulation factors. In LIC, localized activation of the coagulation cascade occurs due to factors such as endothelial damage or inflammation. This localized coagulation can lead to the release of pro-coagulant factors, such as tissue factor (TF) and von Willebrand factor (vWF), into the circulation (5). When these factors are released in significant amounts, they can trigger widespread activation of the coagulation cascade throughout the body, leading to the formation of microthrombi in various organs. Meanwhile, As localized thrombi form in LIC, there is an increased consumption of platelets and coagulation factors in that area (6, 7). This consumption coagulopathy can lead to a state where the remaining coagulation factors are insufficient to maintain hemostasis, resulting in a paradoxical bleeding tendency. This is a hallmark of DIC, where the activation of coagulation leads to both thrombosis and bleeding (6).

VMs are not all evident at birth, and likely to develop fast along with body growing in childhood (8). Especially intramuscular VMs were less likely to present at birth, grow fast with muscle (9), leading to more frequently painful and contained more phlebolith (10). Moreover, haemostasis balance in children also different from that in adults. The concept of “developmental hemostasis” is employed to describe the development process of hemostasis from neofetal stage to adulthood (11, 12). Fibrinogen, FVIII/VWF would go through a U-shaped curve and increase to reach adult values (12). This may lead children with VMs more vulnerable to LIC, suffering from a “not-fully developed” coagulation system. Platelet function also developed with age in children (13) and may account for pediatric LIC in VMs.

Preoperative and postoperative low-molecular-weight heparin (LMWH) administration is considered essential and crucial (14–16). LMWH can stop the pain due to thrombosis, lower D-dimer levels, improve the hematological status and prevent DIC (17, 18). Usually, LMWH should be used at least one week before any interventional procedure, such as surgery or sclerotherapy (17, 19). As for VM children with LIC, D-dimer levels and fibrinogen level were hard to return to normal, which may take weeks.

**Figure 1:**
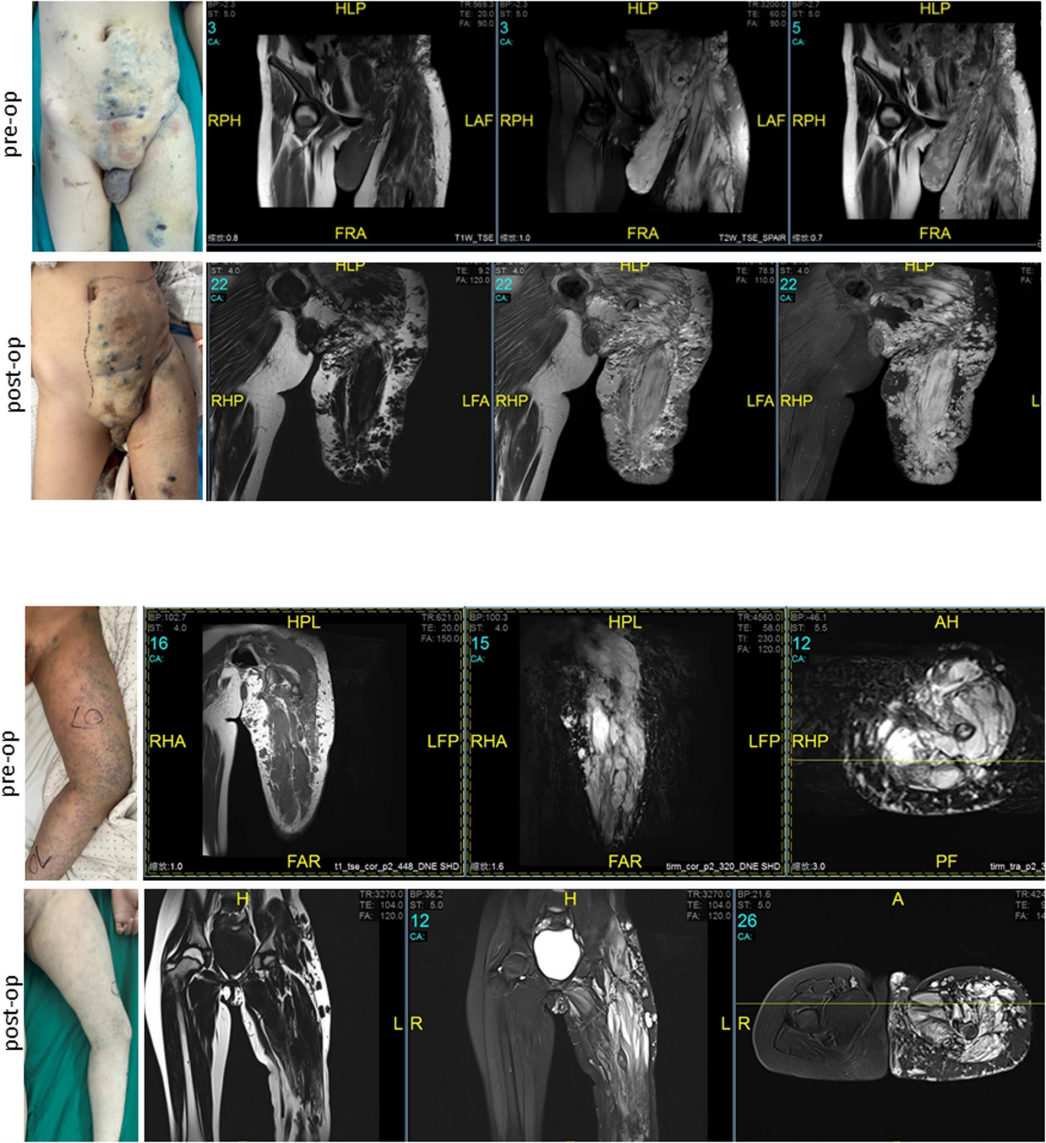
Comparison between pre-treatment and post-treatment from two typical patients.

We retrospectively analyzed 206 pediatric patients (aged < 18 years) with venous malformations who underwent sclerotherapy at Shandong Provincial Hospital Affiliated to Shandong First Medical University between August 2018 and August 2025. Among them, 116 cases had complete laboratory test results and follow-up data. The relationships between age, gender, lesion location and the occurrence of LIC were analyzed. Interventional sclerotherapy was used to treat venous malformation lesions. Patients with preoperative hypofibrinogenemia received fibrinogen therapy postoperatively, and the improvement of LIC after treatment was observed, as shown in Figure 1. We treated with sclerotherapy and LWMH, safe and effective.

## 2 Methods

All patients with venous malformations treated in Shandong provincial hospital vascular surgery department from August 2018 and August 2025 were collected retrospectively. 206 children with venous malformations were evaluated in vascular surgery department of Shandong provincial hospital from 2018 to 2025. Due to lack of clinical data, such as blood coagulation tests or complete medical imaging data from our center and without at least one outpatient follow-up, 90 patients were excluded to avoid bias. The rest 116 patients were further selected with complete laboratory test results and follow-up data (*n* = 116). And children with other d-dimer-elevating diseases were excluded. We obtained data including sex, age at presentation, lesion location, coagulation and fibrinolysis parameters, ultrasound imaging and magnetic resonance imaging(MRI) from the patients records. Our study was approved by research ethical committee from Shandong provincial hospital.

Laboratory analyses included plasma prothrombin time (HemosIL RecombiPlasTin 2G, Werfen Instrumentation Laboratory Co.), fibrinogen(Clauss method, HemosIL Fibrinogen-C XL, Werfen Instrumentation Laboratory Co.), D-dimer (immunoturbidimetric assay, HemosIL D-Dimer HS 500, Werfen Instrumentation Laboratory Co).

Sclerotherapy protocol and LMWH regimen were performed according to related guidelines and expert consensus. Multi-point puncture were used and both reflux of venous blood and DSA angiogram with injection of Iodixanol (Visipaque, GE Healthcare Ireland Limited) to illustrate lesion imaging features and Puig classification. Polidocanol (less than 2 mg/kg), ethanol (0.5 mL/kg) and Glubran-2 (for Puig III or IV, GalenMedical, Italy) were sequentially administrated to make sure as many as lesions covered. LMWH (1 mg/kg once daily) was administrated after LIC diagnosis, while those patients with active bleeding underwent sclerotherapy first and then received LMWH administration, which lasts for 2 weeks and replaced with rivaroxaban (10 mg once daily) for the following 1–2 months when necessary and elder than 10-year old.

The statistical analyses was performed using SPSS 25.0. For the calculation of correlations between coagulation markers, power transformations were used for non-parametric variables. Parametric and transformed non-parametric variables were compared with Pearson's correlation analysis (r) and non-parametric variables with Spearman's rank correlation coefficient (*ρ*). Chi-square test or Fisher's exact test served for paired comparisons between clinical characteristics (LIC diagnosis, location, age, gender) and coagulation markers. Mann–Whitney U-test was used for paired comparisons of non-parametric variables. We considered *p*-values < 0.05 as statistically significant.

## 3 Results

116 children with venous malformations were evaluated in vascular surgery department of Shandong provincial hospital from 2018 to 2025. The median age of our patients was 11 (average 10.95 years). for 54 male children with VM, median age of presentation was 11 years (average 10.22), likewise for 62 female children, that was 12.5 years (average 11.58). The lesions in the 116 patients were localized in the following anatomical regions: head and neck (*n* = 11), trunk (*n* = 11), extremities (*n* = 87), and multiple locations (*n* = 7).

In the multivariate analysis, the results confirmed that sex, age or lesion location were not statistically significantly associated with LIC occurrence.

55 children with VMs (47.4%) had elevated D-dimer levels (0.500 µg/mL) at clinic. To exclude the bias from coagulation examination, we set the cutoff for LIC diagnosis up to 0.60 µg/mL. Thus, 51 children (44.8%) were diagnosed as LIC. And these 51 children with LIC were further classified into minor (*n* = 38 74.5%), moderate (*n* = 11 21.6%), severe group (*n* = 2 3.9%) (Table 2).

Considering limited severe LIC cases, we combined the moderate group and severe group to analyze sex, age or lesion location relationship with LIC severity in VM children (Table 3). Although, female children with LIC has a higher proportion (33.3%) of moderate or severe LIC and male ones has that of only 16.7%, the difference was found not significant. Age or lesion location difference in the two group was also not significant.

There are notable gender-related age differences among children with VM (Table 4). In the low-age group (0–6 years), there are more male patients, with 7 males and 3 females. From 7 to 12 years old, although there are still more male patients (10 males and 9 females), the gap narrows. In the high-age group (13–17 years), female patients outnumber male patients, with 15 females and 5 males. The possible reasons are that young boys have higher levels of physical activity, greater intensity, and a higher probability of trauma compared to girls. For older girls, the increased secretion of estrogen during puberty may lead to a higher probability of symptom occurrence. Statistical analysis shows a significant difference (*P* = 0.040, fisher test).

## 4 Discussion

Our retrospective analysis of 116 pediatric patients with venous malformations (VMs) provides robust clinical evidence that localized intravascular coagulopathy (LIC) is not a rare epiphenomenon but a pathobiologically integral feature of pediatric VMs, affecting nearly half (44.8%) of symptomatic children—mirroring adult prevalence yet carrying distinct developmental and therapeutic implications (20). Crucially, this high incidence was observed despite the absence of systemic triggers (e.g., sepsis, major trauma, or active malignancy), underscoring that the VM lesion itself functions as a chronic, autonomous nidus of coagulation dysregulation—a “living thrombogenic organ” sustained by endothelial dysfunction, stasis-induced hypoxia, and aberrant expression of tissue factor (TF), von Willebrand factor (vWF), and thrombin. This intrinsic prothrombotic microenvironment is further amplified in children by developmental hemostasis: the age-dependent immaturity of anticoagulant pathways (e.g., lower antithrombin III and protein C activity), delayed maturation of fibrinolytic capacity, and platelet hyperreactivity in early childhood—all converging to lower the threshold for LIC initiation and propagation (9, 15, 21).

The lack of statistical association between LIC occurrence and sex, age, or anatomical location (Tables 1, 3) challenges conventional assumptions about demographic or topographic risk stratification. Instead, our data support a lesion-intrinsic, rather than patient-intrinsic, driver of LIC severity. This is powerfully illustrated by the observation that all three cases of severe LIC (D-dimer >20 µg/mL) occurred in patients with extensive, multilevel VMs spanning ≥2 joint planes—particularly involving deep musculature or compartmentalized spaces (e.g., thigh + pelvis, calf + foot). Such lesions create large, low-flow vascular lakes where erythrocyte trapping, fibrin deposition, and platelet activation occur continuously, generating a self-perpetuating cycle of thrombin generation and factor consumption (19). In contrast, small, superficial, or isolated VMs—even when biochemically associated with mild D-dimer elevation—rarely progressed to clinically significant coagulopathy. This suggests that LIC severity is less a function of where the VM lies and more a function of how much functional vascular surface area it presents to the circulation, a concept we term the thrombogenic burden hypothesis. It follows that imaging-based quantification—not just qualitative description—of VM volume, flow dynamics (via dynamic contrast-enhanced MRI), and phlebolith density may prove more predictive of LIC risk than traditional anatomic classification (22, 23).

**Table 1:** Comparison between normal and LIC groups in 116 VM children.

Characteristics	Normal	LIC	Chi-suqare
Sex			0.544
Male	34 (54.8%）	28 (45.2%)	
Female	30 (55.6%)	24 (44.4%)	
Age			0.496
0–6	9 (45.0%)	11 (55.0%)	
7–12	29 (60.4%)	19 (39.6%)	
13–17	26 (54.2%)	22 (45.8%)	
Lesion location			0.158
Head and neck	5 (45.5%)	6 (54.5%)	
Trunk	4 (36.4%)	7 (63.6%)	
Extremities	53 (60.9%)	34 (39.1%)	
Multiple locations	2 (28.6%)	5 (71.4%)	

**Table 2:** Patient characteristics of 51 VM children with LIC (D2 ≥ 0.6 mg/L).

Characteristics	*N* (%)
Sex	
Male	24 (47.1%)
Female	27 (52.9%)
Age	
0–6	11 (21.6%)
7–12	18 (35.3%)
13–17	22 (43.1%)
Lesion location	
Head and neck	6 (11.8%)
Trunk	7 (13.7%)
Extremities	33 (64.7%)
Multiple locations	5 (9.8%)
LIC classification	
Minor (0.6 mg/L＜D2＜5 mg/L)	38 (74.5%)
Moderate (5 mg/L＜D2＜20 mg/L)	11 (21.6%)
Severe (D2＞20 mg/L)	2 (3.9%)

**Table 3:** No significant differences in VM children sex age or lesion location.

Characteristics	Minor LIC	Moderate or severe LIC	Chi-suqare
Sex			0.149
Male	20 (83.3%）	4 (16.7%)	
Female	18 (66.7%)	9 (33.3%)	
Age			0.208
0–6	10 (90.9%)	1 (9.1%)	
7–12	11 (61.1%)	7 (38.9%)	
13–17	17 (77.3%)	5 (22.7%)	
Lesion location			0.097
Head and neck	4 (66.7%)	2 (33.3%)	
Trunk	7 (100%)	0 (0%)	
Extremities	25 (75.8%)	8 (24.2%)	
Multiple locations	2 (40.0%)	3 (60.0%)	

**Table 4:** Gender differences in VM children age.

Age group	*n*	Male	Female	*P*
0–6	10	7	3	0.040 (fisher test)
7–12	19	10	9	
13–17	20	5	15	

A critical translational insight emerging from our cohort is the therapeutic primacy of lesion eradication over anticoagulation alone. While LMWH effectively suppresses systemic coagulation cascade amplification and mitigates microthrombosis, it does not address the root cause: the persistent, abnormal endothelium and stagnant blood pool within the VM. Two patients initially managed conservatively with LMWH monotherapy experienced progressive hypofibrinogenemia and recurrent pain despite normalized D-dimer, only achieving durable remission after subsequent sclerotherapy. Conversely, the two patients with severe LIC who underwent urgent sclerotherapy followed by immediate post-procedural fibrinogen replacement and LMWH demonstrated rapid normalization of fibrinogen (<72 h) and D-dimer (<5 days), with no bleeding or thrombotic complications. This sequence—targeted ablation first, then physiological correction and anticoagulant bridging—provides a new choice different from the former treatment. It aligns mechanistically with the understanding that sclerotherapy, by obliterating the thrombogenic surface, removes the continuous stimulus for TF release and thrombin generation; subsequent LMWH prevents rebound hypercoagulability during the acute inflammatory phase of vessel wall necrosis and repair. For severe LIC patients which may suffer from DIC, we suggest that cryoprecipitate infusions or fibrinogen supplement may play a greater role than low-molecular-weight heparin， because those patients often have active bleeding. Although the use of low-molecular-weight heparin can correct coagulation disorders, bleeding may exacerbate and prolong the course of the disease.

Furthermore, our findings reinforce the clinical utility of LIC biomarkers as real-time surrogates of disease activity and treatment response. We observed a striking temporal concordance: reductions in D-dimer and restoration of fibrinogen consistently preceded and paralleled subjective symptom improvement—particularly resolution of spontaneous pain and reduction in swelling. Notably, in patients with minor LIC, symptom relief occurred before full biochemical normalization, suggesting that even partial reduction in thrombin burden alleviates neurovascular irritation and interstitial edema. This supports the integration of serial coagulation monitoring—not merely as safety surveillance—but as a dynamic therapeutic endpoint, guiding decisions on LMWH duration, need for repeat sclerotherapy, or transition to maintenance therapy.

Finally, our study exposes a critical gap in clinical awareness: LIC remains under-recognized as a sentinel sign of occult VM. All two children diagnosed with severe LIC were initially evaluated for unrelated emergencies—femoral fracture, and nephrotic syndrome—where profound coagulopathy was attributed to the acute condition until VM was incidentally identified on cross-sectional imaging. This diagnostic delay resulted in unnecessary invasive procedures (e.g., exploratory laparotomy), inappropriate transfusions, and prolonged ICU stays. We therefore advocate for mandatory coagulation screening (D-dimer + fibrinogen) in any child presenting with unexplained, persistent coagulopathy—especially when accompanied by soft-tissue swelling, chronic pain, or phleboliths on radiography—regardless of overt vascular lesion visibility. Early suspicion and MRI confirmation of VM could transform management from reactive crisis intervention to proactive, minimally invasive lesion control.

In conclusion, LIC in pediatric VMs is a developmentally amplified, lesion-driven coagulopathy best managed through a precision ablative strategy: risk-stratified by thrombogenic burden (not anatomy), treated with timely sclerotherapy as the cornerstone, and supported by judicious, time-limited LMWH administration. Future work must validate imaging-based thrombogenic burden scoring systems, define optimal LMWH dosing algorithms calibrated to pediatric pharmacokinetics and fibrinogen kinetics, and prospectively test whether early intervention in asymptomatic but biochemically active (e.g., D-dimer >1.0 mg/L) VMs prevents progression to severe LIC—a true opportunity for secondary prevention in vascular malformation medicine.

## 5 Conclusion

The incidence of LIC complicating venous malformations in children is similar to that in adults, and it is more common in older girls. Sclerotherapy can effectively relieve symptoms caused by venous malformations but has little short-term effect on LIC. For LIC patients with hypofibrinogenemia, sclerotherapy combined with fibrinogen transfusion can rapidly correct hypofibrinogenemia.

## Data Availability

The raw data supporting the conclusions of this article will be made available by the authors, without undue reservation.
